# Robustness of genome-wide scanning using archived dried blood spot samples as a DNA source

**DOI:** 10.1186/1471-2156-12-58

**Published:** 2011-07-04

**Authors:** Mads V Hollegaard, Jakob Grove, Jonas Grauholm, Eskil Kreiner-Møller, Klaus Bønnelykke, Mette Nørgaard, Thomas L Benfield, Bent Nørgaard-Pedersen, Preben B Mortensen, Ole Mors, Henrik T Sørensen, Zitta B Harboe, Anders D Børglum, Ditte Demontis, Torben F Ørntoft, Hans Bisgaard, David M Hougaard

**Affiliations:** 1 Department of Clinical Biochemistry and Immunology, Statens Serum Institut, Ørestads Boulevard, Copenhagen, DK-2300, Denmark; 2 Department of Human Genetics, University of Aarhus, Wilhelm Meyers Allé, DK-8000, Aarhus, Denmark; 3 Bioinformatics Research Centre, University of Aarhus, C.F. Møllers Allé, DK-8000 Aarhus, Denmark; 4 AROS Applied Biotechnology AS. Science Park Skejby, Brendstrupgaardvej, DK-8200, Aarhus, Denmark; 5 Copenhagen Prospective Studies on Asthma in Childhood, Copenhagen University Hospital, Ledreborg Allé, DK-2820, Gentofte, Denmark; 6 Department of Clinical Epidemiology, Aarhus University Hospital, Olof Palmes Allé, DK-8200, Aarhus, Denmark; 7 Department of Infectious Diseases, Hvidovre Hospital, Hvidovre, Denmark; 8 Faculty of health Sciences, University of Copenhagen, Blegdamsvej, DK-2200, Copenhagen, Denmark; 9 The National Centre for Register Based Research, Aarhus University, Taasingegade, DK-8000, Aarhus, Denmark; 10 Centre for Psychiatric Research, Aarhus University Hospital Risskov, Skovagervej. DK-8240, Aarhus, Denmark; 11 Department of Bacteriology, Mycology, and Parasitology, Statens Serum Institut, Ørestads Boulevard, DK-2300, Copenhagen, Denmark; 12 Department of Molecular Medicine, Aarhus University Hospital Skejby, Brendstrupgårdsvej, DK-8200, Denmark

## Abstract

**Background:**

The search to identify disease-susceptible genes requires access to biological material from numerous well-characterized subjects. Archived residual dried blood spot (DBS) samples, also known as Guthrie cards, from national newborn screening programs may provide a DNA source for entire populations. Combined with clinical information from medical registries, DBS samples could provide a rich source for productive research. However, the amounts of DNA which can be extracted from these precious samples are minute and may be prohibitive for numerous genotypings. Previously, we demonstrated that DBS DNA can be whole-genome amplified and used for reliable genetic analysis on different platforms, including genome-wide scanning arrays. However, it remains unclear whether this approach is workable on a large sample scale. We examined the robustness of using DBS samples for whole-genome amplification following genome-wide scanning, using arrays from Illumina and Affymetrix.

**Results:**

This study is based on 4,641 DBS samples from the Danish Newborn Screening Biobank, extracted for three separate genome-wide association studies. The amount of amplified DNA was significantly (P < 0.05) affected by the year of storage and storage conditions. Nine (0.2%) DBS samples failed whole-genome amplification. A total of 4,586 (98.8%) samples met our criterion of success of a genetic call-rate above 97%. The three studies used different arrays, with mean genotyping call-rates of 99.385% (Illumina Infinium Human610-Quad), 99.722% (Illumina Infinium HD HumanOmni1-Quad), and 99.206% (Affymetrix Axiom Genome-Wide CEU). We observed a concordance rate of 99.997% in the 38 methodological replications, and 99.999% in the 27 technical replications. Handling variables such as time of storage, storage conditions and type of filter paper were shown too significantly (P < 0.05) affect the genotype call-rates in some of the arrays, although the effect was minimal.

**Conclusion:**

Our study indicates that archived DBS samples from the Danish Newborn Screening Biobank represent a reliable resource of DNA for whole-genome amplification and subsequent genome-wide association studies. With call-rates equivalent to high quality DNA samples, our results point to new opportunities for using the neonatal biobanks available worldwide in the hunt for genetic components of disease.

## Background

Identifying genetic effects in complex disorders usually requires genome studies in large cohorts. Access to DNA from well-characterized patients and healthy controls represents a major bottleneck. This problem may be circumvented by using archived residual blood samples from newborn screening programs, which encompass the entire population under a certain age in several countries. The blood is usually collected by heel-prick and applied to special filter paper; a proven robust and convenient medium for transport and storage [1]. Storage policies for residual neonatal dried blood spot (DBS) samples vary internationally, but several countries store residual samples in repositories for research purposes [2-8]. Stored DBS samples combined with relevant clinical information from medical registries are an ideal resource for large studies representing an entire population under a given age without selection bias. In addition, availability of previously collected samples allows substantial savings in research-related costs and time.

The Danish Neonatal Screening Biobank (DNSB) contains nearly two million DBS samples collected from almost every Dane born after 1981. It has recently been updated to meet new general guidelines for the establishment and operation of biobanks [9]. Approval from the Scientific Ethical Committee System, the Data Protection Agency, and the DNSB Steering Committee is needed to obtain access to samples for research.

In Denmark, all citizens have a unique personal identification number used in all public registration systems, including the DNSB. Denmark also has a well-established public health care system with equal treatment offered to all citizens. These resources allow researchers to study the entire country as a cohort, and make the DNSB an ideal resource for studying common and complex genetic diseases in Caucasians [10].

A major challenge using DBS samples for genetic studies is the small amount of blood available in a spot. The amount of genomic DNA (gDNA) that can be extracted from a 3.2-mm punch of a DBS sample is approximately 60 ng [11]. In general, only one or two 3.2-mm punches per DBS sample can be reserved for a given project, limiting screening to only a few single nucleotide polymorphisms (SNP). This obstacle may be overcome by whole-genome amplification (WGA) of the DNA. Previous studies have used whole-genome amplified DNA (wgaDNA) for genotyping with some success, but in most cases, only a limited number of polymorphisms could be tested [11-17].

Here we describe genome-wide association studies (GWAS) using DBS samples from the DNSB. Storage time, storage conditions, and type of filter paper used for DBS collection were evaluated to determine their effects on the amount of amplified wgaDNA material obtained from each sample. The effects of these variables on genotype call rates in three studies, using three different types of array, running on either Illumina or Affymetrix genotyping platforms were also examined.

## Methods

### Subjects

Our 4,641 subjects were obtained from three case-control GWAS studies. The first study, GEMS (Genomic Medicine for Schizophrenia), called "610k" in this manuscript, included 1,808 DBS samples stored from 1981-1996. The purpose was to identify genetic regions associated with schizophrenia (Ethical Approval no.: 20020020; Data Protection Agency no.: 2002-41-2059). The second study, "Omni1", provided 1,283 DBS samples stored from 1982-2006, and was undertaken to examine the role of genetics in Meningococcal and Pneumococcal infections (Ethical Approval nos. 20060008 and HB-2007-085; Data Protection Agency nos. 2005-41-6012 and 2007-41-0229). The third study, "Axiom", aimed to identify genetic variations associated with asthma, and included DBS samples stored from 1982-2006 (Ethical Approval no. HB-2008-103; Data Protection Agency no. 2008-41-2622). All studies were approved by the DNSB Steering Committee. The current study was conducted as an anonymous register study.

### DNA extraction, whole-genome amplification, and SNP genotyping

Two 3.2-mm disks were punched from each DBS sample, and protein was removed as previously described [18]. Genomic DNA was hereafter extracted using the Extract-N-Amp kit (Sigma-Aldrich). To attenuate possible unequal amplification of alleles, WGA was carried out in triplicate using the REPLI-g mini kit (Qiagen). The concentration of wgaDNA was estimated using Quant-IT PicoGreen dsDNA Reagent (Invitrogen). The three studies, "610k", "Omni1" and "Axiom" used an Infinium Human610-Quad chip array (Illumina), an Infinium HD HumanOmni1-Quad chip array (Illumina) and an Axiom Genome-Wide CEU Array chip (Affymetrix), respectively. wgaDNA samples were normalized to 60 ng/μL prior to genome wide scanning (GWS) of SNP genotypes. Samples with genotyping call rates (GCR) below 97% but above 95% were rerun in the Illumina-based studies without reamplifying the gDNA samples, under the assumption that the low call-rates stemmed from a technical issue. Samples with GCRs below 95% were re-amplified before re-genotyping. Both technical replicates (same wgaDNA genotyped twice) and methodological replicates (same sample of WGA used in two separate reactions and genotyped separately) were included in the two Illumina studies. "610k" included six methodological and 11 technical replicates, "Omni1" included 32 methodological and 16 technical replicates, and "Axiom" had no replicates.

### Statistical evaluation

To evaluate the sample processing we pooled the DBS samples included in the three GWAS studies. Pooling samples was statistically sound as all samples were treated identically up to the step before choosing the SNP genotyping array platform and technology, but possible sample effects were tested statistically. We used a linear regression model to test for interaction between the included variables: years of storage counting from 1981 (years), storage conditions (condition: 0 (+4°C, 1981-1987), 1 (-20°C, 1988-present)), type of filter paper (filter: 0 (S&S2992, 1981-2000), 1 (S&S903, 2001-present)) and the wgaDNA concentration.

The GCR was used for evaluating the array efficiency and sensitivity to biobank variables. To meet the criteria of being normally distributed, the GCR was transformed using a zero-skewness log (resulting in log(1-GCR), the logarithm of the failure rate). The effect of the years of storage, type of filter paper, and storage conditions on the transformed GCR was analysed in the three studies individually using a linear regression interaction model. A bivariate linear regression model was used to evaluate the effect of the wgaDNA concentration on the GCR. STATA MP11 software (StataCorp LP, TX, USA) was used for the statistical analyses.

## Results

### Subjects

A total of 4,641 subjects from the three GWAS disease studies were used to evaluate the use of DBS samples for genetic studies. "610k" included 1,808 samples stored for a mean of 23.9 years (range: 14-28 years; standard deviation (SD): 2.8 years; 45.5% female and 54.5% male). "Omni1" was based on 1,283 samples stored for a mean of 15.7 years (range: 4-28 years; SD: 6.2 years; 43.6% female and 56.4% male). "Axiom" included 1,550 samples stored for a mean of 16.5 years (range: 4-28 years; SD: 6.5 years; 31.8% female and 68.2% male).

### Biobank evaluation

**Figure 1:**
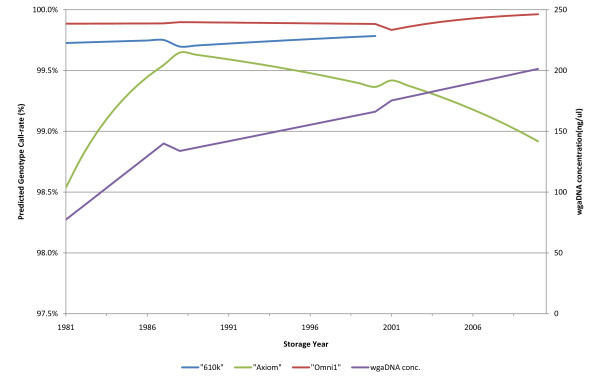
**Regression model predicted wgaDNA concentrations and GCR by storage year**. The predicted effect of the year of initial storage on the GCR in the three case-control studies (blue: 610k; green: Axiom; red: Omni19) are shown on the primary (left) y-axis. The secondary (right) y-axis shows the predicted wgaDNA concentration (purple line) over the year of initial storage.

As the three studies used the same sample preparation process, we were able to pool their results to evaluate the method. As both storage conditions and filter paper are time dependent, bivariate analysis was not appropriate. Results of the multiple regression models summarised in Table 1. A graphical representation of the model is shown in Figure 1 (wgaDNA concentration graph). The mean wgaDNA concentration (Total volume: 180 μL) of the 4,597 DBS samples was 135 ng/μL, ranging from 5-442 ng/μL, and with a standard deviation of 51 ng/μL. The type of filter paper did not significantly affect the wgaDNA concentration, nor did it significantly change the effect of time of storage. However, the wgaDNA concentration was significantly lower in the older samples, with each year of storage accounting for an average loss of 10.4 ng/μL per year. The negative effect of time of storage was significantly decreased if the samples were stored at -20°C shortly after reception; the rate of degradation reduced to 2.67 ng/μL per year.

**Table 1:** Effect of storage year, storage conditions, and type of filter paper on the wgaDNA concentration.

	**Coef**.	95%CI	**Std.Err**.	p-value
Year	10.4	(9.39; 11.5)	0.531	0.000
Condition	37.6	(30.5; 44.7)	3.62	0.000
Condition*Year	-7.74	(-8.87; -6.61)	0.577	0.000
Filter Paper	2.68	(-81.9; 87.3)	43.2	0.950
Filter Paper*Year	0.193	(-3.63; 4.01)	1.95	0.921
Constant	77.4	(73.6; 81.2)	1.93	0.000

Multiple linear regression model testing for interaction between the included variables: storage year (Year), storage conditions (Condition: +4°C vs. -20°C), type of filter paper (Filter Paper: S&S2992 vs. S&S903) and the wgaDNA concentration. Coef.: regression coefficient. 95%CI: 95% Confidence interval. Std.Err.: Standard error. A p-value of 0.000 is to be interpreted as being below 0.0001.

### Technical evaluation

In the "610k" and "Omni1" studies, 60 and 18 samples, respectively, failed to meet our predefined GCR criterion of > 97% and were requeued (Table 2). Eleven samples in the "610k" study and five in the "Omni1" study failed to reach the CGR criterion in the second run. A total of 98.8% of samples in the "610k" study and 99.6% in the "Omni1" study were successfully genotyped (Table 2). Method samples (the same gDNA sample amplified twice and genotyped separately) were repeated six times in "610k" and 32 times in "Omni1", with concordance rates of 99.999 and 99.994%, respectively. Technical samples (the same wgaDNA sample run twice on separate chips) were replicated 11 times in "610k" and 16 times in "Omni1", with concordance rates of 99.950 and 99.998%, respectively.

**Table 2:** Technical evaluation of the "610k", "Omni1", and "Axiom" studies.

	"610k"	"Omni1"	"Axiom"
Subjects	1808 (100)	1283 (100)	1550 (100)
Positive amplification	1799 (99.5)	1283 (100)	1550 (100)
Successful genotyping	1778 (98.8)	1278 (99.6)	1530 (98.7)
Requeue	60 (3.34)	18 (1.40)	
Successfully requeued	39 (65.0)	13 (72.2)	
Methodological replicates	6 (99.999)	32 (99.994)	
Technical replicates	11 (99.950)	16 (99.998)	

Number and percentage (%) of included subjects. Subjects: number of subjects initially included. Positive amplification: number of subjects with a successful WGA reaction (conc. > 30 ng/ul). Requeue: gDNA samples failing to reach initial successful genotyping criteria that were whole-genome amplified and genotyped a second time. Successfully requeued: The number of requeued samples successfully genotyped. Methodological replicates: the same gDNA sample amplified twice and genotyped separately. Technical replicates: the same wgaDNA sample run twice on separate chips.

### Array evaluation

The three GWAS studies were evaluated separately as their performance was significantly different (data not shown), likely because different array types were used on different genotyping platforms (Affymetrix and Illumina).

The mean GCRs in the three studies were: "610k", 99.385385% (range: 47.129-99.933%; GCR 5th percentile: 98.609%); "Omni1", 99.722 (range: 55.685-99.974%; GCR 5th percentile: 99.522%); and "Axiom", 99.206% (range: 89.313-99.890%; GCR 5th percentile: 98.140%).

Considering that wgaDNA concentration is an intermediate variable between GCR and years of storage and storage conditions (Table 1), we did not include it in the model for GCR, but analysed it as a single factor using linear regression. An increasing wgaDNA concentration was found to positively affect the GCR in the Omni1 (P < 0.001), and Axiom (P = 0.013) arrays, whereas the 610k array (P = 0.228) was not significantly affected. The multiple-linear regression models with interactions showed: storage conditions, years of storage and filter paper type were significantly associated to the GCR in at least one of the three arrays (Table 3). For the Omni1 array we saw a significant association between the type of filter paper and the GCR, suggesting that samples collected on S&S2992 filter paper perform better than samples collected on S&S903 filter paper. Furthermore, the "filter*years" interaction variable indicated that the GCR decreases more rapidly over time on S&S903 paper (as is also indicated by the steeper curve in Figure 1). Samples genotyped with the Axiom array, stored at -20°C had significantly higher GCRs than samples stored at +4°C, but the effect of storage years on GCR was reversed when storing at -20°C. "610k" did not include samples collected on S&S903 filter paper, and neither storage time nor conditions affected the GCR.

**Table 3:** The effect of biobank-related variables on log(1-GCR) in three GWS arrays.

		"610k"			"Omni1"			"Axiom"	
	**Coef**.	95%CI	p-value	**Coef**.	95%CI	p-value	**Coef**.	95%CI	p-value
Year	-0.016	(-0.040; 0.009)	0.204	-0.004	(-0.080; 0.072)	0.921	-0.195	(-0.238; -0.151)	0.000
Condition	25.1	(-107; 157)	0.710	-30.3	(-185; 124)	0.701	-484	(-574; -394)	0.000
Condition*Year	-0.013	(-0.079; 0.054)	0.712	0.015	(-0.063; 0.093)	0.703	0.243	(0.198; 0.289)	0.000
Filter paper				357	(108; 606)	0.005	-40.1	(-222; 142)	0.666
Filter paper*Year				-0.178	(-0.303; -0.054)	0.005	0.020	(-0.071; 0.111)	0.667
Constant	25.3	(-23.0; 73.6)	0.304	0.798	(-150; 152)	0.992	381	(294; 468)	0.000

Included variables: Storage year (Year), storage conditions (Condition: +4°C vs. -20°C), type of filter paper (Filter paper: S&S2992 vs. S&S903) and the interaction variables Condition*Year, and Filter Paper*Year, and the GCR. Coef.: regression coefficient. 95%CI: 95% Confidence interval. A p-value of 0.000 is to be interpreted as being below 0.0001.

## Discussion

Dried blood spot samples are being collected and stored in biobanks for diagnostic and research purposes worldwide. In several countries this has been common practice for several decades. We previously showed that DBS samples from the DNSB can be used to generate reliable genetic results using the Illumina genome-wide scanning technology, but this evaluation was restricted to relatively few samples [19]. In the current study, which combined results from three recent GWS studies, we found that DBS samples are suitable for large-scale genetic studies [20].

**Figure 2:**
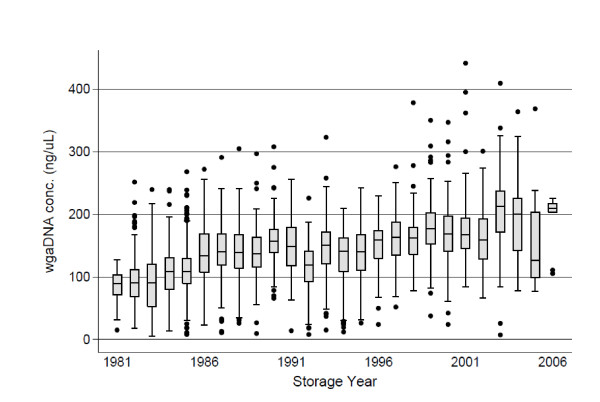
**Effect of years of storage on wgaDNA concentration**. The X-axis represents the year of initial storage of DBS sample, and the y-axis shows the wgaDNA concentration in ng/uL. Black dots represent outlier values, and the adjacent lines the upper and lower adjacent values. The upper and lower hinges represent the75^th ^and 25^th ^percentile values, respectively, and median values are marked inside the box.

Storage time influenced the wgaDNA amplification negatively, with significantly lower concentrations in the older samples (Table 1)(Figure 1)(Figure 2). The concentration was lowest in samples from 1981-82, and slowly increased for samples until 1988-90. Concentrations then remained relatively stable until 2001-02, and then increased again until 2006. From 1981-1988, the policy was to store the samples at +4°C for up to a year before transferring the sample to -20°C, possibly increasing fragmentation of the spot DNA, and lowering the quality of the DNA.

According to our regression model, and as suggested in a previous study [17], increasing years of storage and storage at+4°C negatively affected the wgaDNA concentration (Table 1). Independent of years of storage, the wgaDNA concentration increased when DBS samples were stored at -20°C shortly after reception, thereby increasing the chance of a successful WGS (Table 1). In contrast to a previous finding [17], the more absorbent S&S903 filter paper did not significantly affect the amount of amplified material compared to the less absorbent S&S2992 filter paper (Table 1) [17]. As only 8.6% (396) of the samples were spotted on S&S903 filter paper, we would like to expand this analysis when new studies have provided additional data from S&S903 samples.

The wgaDNA samples in the three studies performed excellently, with mean GCRs greater than 99.2%, and replication concordance rates greater than 99.9% (Table 2). This indicates that gDNA extracted from DBS samples, amplified under suboptimal conditions (gDNA input below 10 ng), can be used as a reliable DNA resource for high-throughput SNP genotyping. With this in mind, we aimed to detect if any biobank-related variables affected the GCR.

In "Omni1" and "Axiom", but not "610k", increasing wgaDNA concentrations increased the GCR. We speculated as to whether the lack of association in "610k" was due to the fact that the study only included samples from 1981-1996, whereas the other studies incorporated samples stored from 1982-2006.

In contrast to what we expected, the less absorbent S&S2992 filter paper had significantly higher GCRs in "Omni1", compared with the absorbent S&S903. The statistical model also indicated that the GCRs in the S&S903 samples of the"Omni1" increased significantly with fewer years of storage, suggesting that the GCRs over time decrease at a higher rate. Overall, relatively few samples were collected on the S&S903 filter paper (196 (~15%) in "Omni1" and 200 (~13%) in "Axiom"), so the significant associations could also be artefacts. Future studies will help us to answer this question.

The storage conditions significantly affected the GCR in the "Axiom" study, with the GCR increasing when samples were stored at -20°C. Unexpectedly, the older samples performed better than the more recent samples. We speculate that the difference between the Affymetrix (ligation) and Illumina (single base extension) SNP genotyping approaches may contribute to this. Overall, of the three arrays tested, the Illumina "Omni1" array performed best. Compared with the other arrays, "Omni1" had the highest mean GCR, and the highest sample success rate. It is important to point out that none of the arrays performed poorly, and that the effects of the different variables on the GCR were minimal, even when statistically significant. All three arrays should be considered usable for GWS of DBS samples.

The robustness of the three GWS studies indicates that filter paper is an excellent way to collect and store whole blood samples for later DNA research purposes. Collecting samples on filter paper has several advantages compared with standard venepuncture, including less discomfort for the patient, especially if several samples need to be collected within a short period of time. The relatively small amount of blood taken limits the number of analyses that can be performed, but techniques such as WGA help mitigate these restrictions with regard to DNA based methods. To date, DBS samples have been used for multiplex protein analysis [21], Vitamin D estimation [22], mRNA profiling [23], cytomegalovirus identification [24], and epigenetic methylation testing [25]. These studies, combined with the ability to perform a full genetic SNP profile that we describe here, show that consider DBS biobanks can be considered sources for sample material for future studies of disease. It remains to be seen whether DBS samples can be used for next-generation sequencing, universal epigenetic profiling or detection of copy number variations.

## Conclusion

In summary, we found that DNSB DBS samples constitute a good resource for SNP genotyping and GWS array studies. Samples in neonatal screening biobanks worldwide should be considered an important source of genetic material for future genetic studies. Our results also suggest suggests that new samples for GWS studies can be collected on filter paper with minimal discomfort for patients, potentially higher participation rates, and convenience in collection, shipping, costs, and storage as compared with whole blood obtained by venepuncture. Depending on the array chosen, different variables may marginally affect the GCR, but overall our approach using DBS samples stored for up to 28 years performed as well as good quality DNA from whole-blood samples. Though not significantly affecting the GCR, we emphasize the importance of storing DBS samples at-20°C, to enhance the numbers of biomarkers that can be analysed.

## Authors' contributions

MVH drafted the manuscript, managed the DBS samples and genetic material used in the three studies, analysed the genetic GWS data in the "610k" and "Omni" studies, did the statistical analyses, and interpreted the results. JAG revised the statistical analysis, interpreted data, and revised the manuscript critically. JOG performed the GWS of the "610k" and "Omni" studies and was involved in the quality testing of the arrays. EKM performed the initial analysis of the "Axiom" GWS data, and revised the manuscript critically. KB contributed substantially to the conception and design of the "Axiom" study and took part in the initial analysis of the GWS data. MN and TLB conceived the "Omni" study, and participated in its design and coordination, took part in the initial analysis of the "Omni" GWS results, and revised the manuscript critically. PBM, OM, TFO, and BNP conceived the "610k" study, and participated in its design and coordination. HTS contributed substantially to the conception and design of the "Omni" study, and revised the manuscript critically. ZBH contributed substantially to the conception and design of the "Omni" study. ADB conceived the "610k" study, and participated in its design and coordination, and revised the manuscript critically. DD performed the quality test of the "610k" GWS data, and revised the manuscript. HB conceived the "Axiom" study, and participated in its design and coordination. DMH conceived the "610k" study, participated in its design and coordination. DMH also participated in the coordination of the "Omni" and "Axiom" studies, and critically revised the manuscript. All authors read and approved the final manuscript.
